# Cardiac MRI for evaluation of paravalvular leak after Transcather Aortic Valve Replacement

**DOI:** 10.1186/1532-429X-15-S1-P60

**Published:** 2013-01-30

**Authors:** Chesnal D Arepalli, Raul R Blanco, Mihir Kanitkar, John N Oshinski, Vasilis C Babaliaros, Peter C Block, Vinod Thourani, Robert A Guyton, Arthur E Stillman, Stamatios Lerakis

**Affiliations:** 1 Cardiology, Emory University, Atlanta, GA, USA; 2 Radiology, Emory University, Atlanta, GA, USA; 3 Cardiothoracic Surgery, Emory University, Atlanta, GA, USA

## Background

The most common complication of Transcathether Aortic Valve Replacement (TAVR) is aortic regurgitation (AR). Typically, this regurgitation is in the mild range, yet in a smaller subset AR could be in the moderate-severe range. Significant regurgitation is usually due to paravalvular leak due to undersizing of the valve or malposition inferiorly into the left ventricular outflow tract or superiorly into the aorta during deployment. Transthoracic Echocardiography (TTE) is first line test for the amount of regurgitation, but can be flawed due to poor acoustic windows and eccentricity of the paravalvular leak. Cardiac MRI (CMR) may be used to assess the aortic regurgitation when there is a discrepancy with echocardiography or imaging of the valve is in question.

## Methods

**Figure 1:**
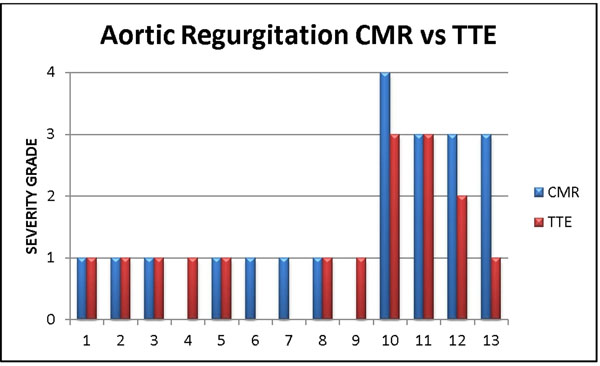
Severity grade of AR after TAVR using CMR and TTE in 13 patients.

We examined 13 patients who underwent TAVR as part of the PARTNER trial and subsequently had a CMR for evaluation of aortic regurgitation (figure 1). Total AR (valvular and paravalvular) was graded by CMR using flow quantitation as 0 (<1% Regurgitant Fraction), 1 (1-29%), 2 (30-39%), 3 (40-49%) and 4 (>50%). Total AR was graded by TTE using color doppler as 0 (none), 1 (trivial or mild), 2 (moderate), 3 (moderate severe) or 4 (severe).

## Results

Typical for PARTNER trial demographics, mean age is 87 +/- 5 years with 11 males. Aortic Regurgitation was predominantly paravalvular. In 12 of the 13 patients TTE was able to accurately assess severity of aortic regurgitation within 1 severity grade as compared to CMR. In 1 patient, echocardiography diagnosed mild AR despite good windows and doppler studies while CMR regurgitant fraction was 45% in the moderate-severe range.

## Conclusions

CMR may provide an accurate assessment of aortic regurgitant fraction after TAVR when physical exam and echocardiographic evidence is noncongruent.

## Funding

None

